# Perceptions of healthcare quality indicators by anesthesiologists in intensive care departments in Ukraine

**DOI:** 10.1186/s12913-025-13922-z

**Published:** 2025-12-23

**Authors:** Maksym Barsa, Valentyna Anufriyeva

**Affiliations:** 1 Departmental Directorate, Anesthesiologist, Anesthesiology and intensive care department in Communal enterprise “Yuri Semenyuk Rivne Regional Clinical Hospital” of Rivne Regional Council, Rivne, Ukraine; 2 Faculty of Healthcare, Social Work and Psychology, School of Management in Healthcare in National University of Kyiv-Mohyla Academy, Kyiv, Ukraine; 3 Ukrainian-Swiss Project “Medical Education Development”, Swiss Tropical and Public Health Institute, Kyiv, Ukraine

**Keywords:** Healthcare, Healthcare quality, Perceived quality, Quality indicators, Healthcare management, In-patient care, Intensive care, Anesthesiology

## Abstract

**Background:**

During the last decade more and more countries plan to reach universal healthcare coverage by 2030. Financial healthcare reform in Ukraine started in 2016, focusing on effective financing and providing access to qualitative medical services to the population. Primary healthcare started to change in 2018 followed by secondary and tertiary care in 2020. In 2022–2023 great challenges emerged because of the full-scale war. The aim of this research is to describe the perception of healthcare quality indicators among anesthesiologists in intensive care units in Ukraine, that might be used to improve the quality assessment system in Ukraine as well as in other countries that have similar healthcare systems or undergo similar processes of changing their specialized care.

**Methods:**

Data were collected in November 2024 - January 2025 applying the survey as the main data collection method. The normality of the distribution of respondents’ age and work experience was assessed by the Kolmogorov-Smirnov test followed by the Mann-Whitney U test. Responses to the open-ended questions were initially categorized according to the established framework and subsequently analyzed using descriptive statistics and binary logistic regression analysis.

**Results:**

In total, 114 filled in questionnaires were received and included in the analysis. Doctors-anesthesiologists tend to perceive quality as a process quality. Among the most important clinical quality indicators for anesthesiologists there are “prevention of thromboembolism” (99.1%), “prevention of ulcer formation” (98.3%), “control of ventilator-associated pneumonia” (98.3%). Non-clinical indicators included “nurse-to-patient ratio” (100%), “availability of a mechanical ventilation weaning protocol” (100%) and “compliance to clinical guidelines” (96.5%). A statistically significant relationship (p˂0.05) between the indicator “Satisfaction of the patient’s relatives with the patient’s stay in the intensive care unit” and the independent variable “owner” was found.

**Conclusions:**

Our research provides new insights into perception of healthcare quality and importance of quality indicators for its assessment among the medical doctors of specialized care. In comparison with the general practitioners focusing on the process quality, the focus of doctors-anesthesiologists lies in the outcome quality. But little consensus about healthcare quality remains to be an issue, as does the necessity to develop and promote a national quality policy and a national quality strategy.

**Supplementary information:**

The online version contains supplementary material available at 10.1186/s12913-025-13922-z.

## Introduction

Over the decade the number of countries planning to reach universal healthcare coverage by 2030 increases [1]. This means the opportunity for all people and communities to use necessary and efficient information, preventive, treatment, rehabilitation and palliative healthcare services of good quality as well as absence of financial difficulties connected with these services [1].

At the same time, it is impossible to ensure the provision of the universal healthcare services only by coexistence of the infrastructure, medical goods and medical service providers. Healthcare services provision also requires attention to quality in the meaning of safe, people-oriented, timely, efficient, effective and integrated care [1]. 

Financial healthcare reform in Ukraine started in 2016. Its focus laid in providing access to qualitative healthcare services to the population and effective financing [2]. Legislative changes were followed by financial and managerial changes in the primary healthcare in 2018 and later in the secondary and tertiary care in 2020. In 2022–2023 great challenges emerged because of the full-scale war.

The shift from traditional budget-line financing of hospitals to a contract-based model was implemented under the principle “money follows the patient.” The healthcare facilities were no longer financed through fixed annual budgets but through payments for specific medical services actually provided to patients based on the quantity and quality of the delivered services, linking funding directly to patient outcomes and institutional performance [2]. Healthcare facilities gained financial autonomy. At the same time, the autonomy pushed healthcare managers, who traditionally were chosen between the medical doctors, to study management. In other words - to improve financial, human resources and strategic decisions in competitive environment [3].

National Health Service of Ukraine (NHSU) was created in the capacity of national payer which has the right to conclude agreements with healthcare providers and guarantees transparency of financing as well as quality of medical care [2]. The system of electronic healthcare (eHealth) was introduced. Electronic prescriptions, referrals and patient records were supposed to create more conveniences both for healthcare providers and for patients.

It was planned that the change in healthcare financing would be profitable for medical doctors as well [4]. Doctors, who attract more patients, provide more services, including more complicated ones, receive higher income, which in its turn stimulates more qualitative work. The more medical doctors manage certain clinical situations, the more they improve their skills, which has a positive effect on the quality of medical services [5]. Also, the competition between the medical doctors motivates them to invest into their skills improvement, equipment, creation of comfortable environment for doctors and patients, introducing modern treatment techniques and improving communication with the patients [6].

Healthcare quality is rather subjective, which complicates its assessment [7]. Perception of quality depends on the views of different stakeholders. For patients quality is associated with comfort, medical personnel attitudes, time of the service provision or clear communications [8]. For healthcare professionals it is associated with compliance to clinical guidelines, treatment effectiveness and patient safety [9]. For policy makers – with compliance to standards, rational use of resources and reaching certain statistical indicators [9].

The provision of qualitative medical care in intensive care units (ICU) requires special attention as the care is provided to patients with the most complicated pathologies and the highest mortality rate [10]. Complex cases, invasive procedures as well as interdependency of several departments and their dependency on teamwork, add to the high risks of mistakes and adverse events [11].

Organization of qualitative medical care in ICU is complicated and multidimentional. It requires application of a conceptual model with relevant quality assessment indicators, including the assessment of structure, process and outcome [12, 13]. At the same time, quality assessment potentially reduces risks of iatrogenic and organizational adverse events for ICU patients [14].

Multidimensional model of quality of medical care by Robert Maxwell defines multidimensionality through classical Donabedian’s structure, process and outcome [7] and the six dimensions of medical care quality (effectiveness, acceptability, efficiency, access, equity, relevance), each having the developed indicators for quality assessment in ICU [15]. Application of the model makes it easier to see what aspects of medical care in ICU require improvement. At the same time, clinical quality assessment is as much important. That is underlined by many researchers who studied clinical indicators of quality. For example, Maartje de Vos et al. [16] developed another model of quality assessment in ICU by defining the list of clinical indicators. Indicators of structure: availability of resuscitators (hours per day), patients to nurses ratio, strategy of treatment mistakes prevention, assessment of patient satisfaction and/or patient family satisfaction. Indicators of process: duration of stay in ICU, duration of mechanical ventilation, proportion of days when all the beds in ICU were occupied and proportion of glucose measurements above 8.0 mmol/l or below 2.2 mmol/l. Indicators of outcome: mortality rate (APACHE II), frequency of bedsores, number of unplanned extubations.

Later on, other models were developed and introduced into practice. For example, Hybrid Healthcare Quality Assessment developed by Tossaint-Schoenmakers et al. (2022) defining 8 clusters and 33 factors of quality [17].

In Ukraine approaches to quality assessment in intensive care units remain largely heterogeneous and fragmented. According to the World Bank report [4], evaluation of quality in hospitals is still often oriented toward administrative control and punishment rather than systematic improvement. In most cases, anesthesiologists and ICU heads rely on institutional or managerial reporting forms rather than structured quality frameworks such as Maxwell’s dimensions or Donabedian’s model. As a result, quality assessment is perceived more as a bureaucratic requirement than as a managerial instrument for continuous improvement.

Perception of quality indicators among medical doctors influences preferences of their use and indirectly influences decision making after their analysis [18]. Medical doctors trust in assessment systems in general and assessment of outcomes predisposes the effectiveness of the managerial interventions. Moreover, the level of acceptance of the quality indicators by medical doctors has an important value for the effectiveness of the healthcare system [19].

Understanding the perception of quality indicators helps to detect the barriers for their application, for example, insufficient understanding, doubts about their scientific justification or lack of trust towards the data source. This helps to adapt educational, managerial or technical interventions [18].

ORCHESTRA research conducted in Brazil in 78 intensive care units shows that the application of clinical indicators and participation of medical personnel influences significantly the clinical outcome, specifically the mortality rate and the resources usage [19].

Another research by Ortiz-Prado et al. [20] shows that effective use of quality indicators requires active participation of medical doctors in the processes of their development and application. It increases the levels of acceptance and influence on clinical practice. The research was conducted in Ecuador among 607 medical doctors who worked in state and private medical facilities. Less than half of the respondents (43%) considered local healthcare system to be efficient, the rest expressed their mistrust to administrative processes and doubts about the value of clinical indicators for quality improvement. At the same time, the lack of participation in managerial decisions was observed, which also influences the level of trust towards the introduced changes.

In the systematic literature review Renker-Darby et al. [21] analyze physicians’ perspective on clinical indicators, including intensive care. The results show that the effectiveness of such indicators depends on including the medical doctors into their development, convenience of their use, meaningful feedback and availability of resources for changes. The indicators of the aspects that are under control of medical doctors have the highest acceptability level. Usage of the indicators as a tool for control or punishment reduces trust to the system and leads to the narrow interpretation of the term “quality”.

Though the importance of quality indicators is well-perceived, the number of studies of the perception of indicators among healthcare professionals in Ukraine remains limited.

Anufriyeva et al. [22] studied the perception of quality and quality assessment tools used by healthcare managers who are at the same time medical doctors and nurses. Data collection took place in April-May 2019. Three hundred and two respondents took part in the research. The results show that primary care professionals perceive quality as compliance to standards, communications with patients and organizational effectiveness. Personal understanding of quality influences preferences in management and assessment tools.

We did not find research on quality indicators perception among medical doctors in intensive care units in Ukraine. It is therefore important to study how medical doctors-anesthesiologists perceive quality indicators especially under the conditions of ongoing war and increased workload on ICU. Strengthening awareness and practical skills in evidence-based quality management among ICU professionals might significantly contribute to the maturity and readiness of the system for future quality management initiatives.

The aim of this research is to describe the perception of healthcare quality indicators among anesthesiologists in intensive care units in Ukraine. We expect that the proposed indicators will be acceptable to anesthesiologists to ensure a comprehensive and equitable assessment of the quality of medical care provided in the ICUs of specialized healthcare facilities. We also hypothesize the presence of a statistically significant relationship between our proposed quality indicators and independent variables, such as gender, experience, job title, level of medical care and the English language proficiency.

The evidence of the perception of healthcare quality indicators among anesthesiologists in intensive care might be used to improve the quality assessment system in intensive care units in Ukraine as well as in other countries that have the similar healthcare systems or undergo the similar processes of changing their specialized care.

## Methods

A study of the perception of indicators of the quality of medical care was conducted among anesthesiologists in intensive care units of specialized medical care institutions in Ukraine. Data collection took place during November 2024 - January 2025 applying the survey as the main data collection method. The survey allows to obtain both subjective assessments and quantitative indicators, which contributes to a comprehensive analysis of the research results [23].

The semi-structured survey was conducted online by means of a questionnaire created in Google Forms. The questionnaire contained four blocks of questions.

Block 1 “Perception of quality, quality assessment tools” consisted of 16 open-ended questions. Respondents described their understanding of the structure, processes, and outcomes of quality care delivery, including its effectiveness, acceptability, efficiency, access, equity, and relevance, as well as the tools used to assess them.

Block 2 “Assessment of the importance of quality indicators of medical care in ICU” included 34 closed questions. Respondents assessed the importance of quality indicators. The Likert scale was used.

Block 3 “Interpretation and perception of indicators” consisted of 15 open-ended questions aimed at exploring respondents’ views on the informativeness, significance, and practical application of clinical and non-clinical quality indicators.

Block 4 “Information about the focus group participant” contained questions about the respondents age, gender, work experience, job title, place of work, and English proficiency.

The detailed formulation of questions can be found in Appendix 1.

Assessment of English proficiency among intensive care physicians was included in the questionnaire to determine access to foreign sources and as an indirect indicator of the level of awareness of the principles of evidence-based medicine. Considering the limited number of up-to-date sources of literature in the Ukrainian language, knowledge of the English language opens access to the latest research, crucial for the quality of treatment. [24, 25].

The questionnaire for anesthesiologists was created based on the Maxwell approach to assessing quality in intensive care units [26] with the addition of key clinical indicators of medical care in the ICU according to De Vos such as the presence of anesthesiologists (hours per day), length of stay in the intensive care unit, number of unplanned extubations, etc. [16].

Maxwell’s approach [26] is a multidimensional conceptual model for assessing healthcare quality that considers both clinical and organizational aspects of service delivery. Within this model, quality in intensive care units is assessed across six key dimensions: effectiveness, acceptability, efficiency, accessibility, equity, and relevance.

Maxwell’s model has been in use across the United Kingdom, Canada, and other countries with well-developed healthcare systems to evaluate the quality of both inpatient and outpatient care. [27]. In particular, Maxwell’s model has been actively employed in Canada’s policy development in the field of healthcare management [28]. It has proven to be a valid and reliable framework that enables the assessment of clinical outcomes, patient perceptions, and resource efficiency, particularly in complex, multifactorial settings such as intensive care units.

The selection of this model as the conceptual framework for our study is justified by its flexibility, adaptability to diverse healthcare contexts, and its comprehensive approach, which enables not only the assessment of the quality of medical service delivery but also the identification of problem areas and potential strategies for improvement.

The relevance and utility of Maxwell and De Vos indicators have been substantiated by numerous studies, highlighting their value in evaluating healthcare quality. Organizational and process indicators such as the participation of a pharmacist during daily rounds [29], the availability of a mechanical ventilation wearing protocol [30], clearly defined criteria for patient admission and transfer, and the implementation of a quality improvement strategy [31] serve as markers of effective healthcare management and alignment with contemporary clinical standards. Pain management protocols, daily sedation interruption guidelines [32], and the use of computerized entry systems of physicians prescriptions are also critical components, as they contribute to reducing the risk of medical errors [33].

Clinical outcomes serve as key indicators of the safety and effectiveness of treatment [34]. Relevant clinical outcomes encompass rates of deep vein thrombosis, pulmonary embolism, pressure injuries, stress ulcers, unplanned extubations, and the need for blood component transfusion. Glucose control is a critical parameter influencing patient prognosis, particularly in intensive care settings [35].

Treatment effectiveness and resource efficiency are assessed using indicators such as surgery cancellations and postponements, ICU readmissions [36], premature discharges, and the duration of mechanical ventilation [37]. Additionally, treatment intensity assessment tools like the Therapeutic Intervention Scoring System (TISS) [38] are applied to quantify the level of care provided.

Finally, retrospective evaluation of care quality is conducted through the review of autopsy findings, which enables assessment of the accuracy of clinical diagnoses and the effectiveness of the therapeutic interventions provided [39].

Our questionnaire was pretested using a focus group consisted of one healthcare administrator and four subject-matter experts. The selection criteria for focus group participants included: i) being an anesthesiologist recognized by peers as an opinion leader, ii) having a minimum of ten years of experience in intensive care, and iii) possessing demonstrated expertise in organizing medical care delivery within intensive care units.

Following the focus group discussion, the questionnaire was finalized by incorporating additional items on quality indicators highlighted by the experts as particularly important. Those included: 1) availability of a closed or open ventilator system, 2) availability of educational materials for patients and their relatives 3) monitoring adherence to the pain management protocol 4) incidence of ventilator-associated pneumonia (VAP), 5) frequency of delirium among ICU patients, 6) empathy demonstrated by medical staff.

Table 1 Presents the final set of quality indicators developed through a synthesis of Maxwell’s framework [15] with the addition of key clinical indicators of medical care in the ICU according to De Vos et al. [16] and additional items on quality indicators highlighted by the experts.

**Table 1:** Quality assessment in intensive care units

	Structure	Process	Outcome
Effectiveness	Doctor-to-patient ratio	Shift duration**	Comparison of survival rates among similar cases across comparable intensive care units
	Nurse-to-patient ratio	Compliance to protocols**	Complication rate and spread of infections*
	Assistant-to-patient ratio	Compliance to evidence-based practice standards**	Ventilator-associated pneumonia*
	Approval and availability of necessary equipment in accordance with the specific type of medical care provided	Open or closed systems for tracheobronchial tree (TBT) sanitation*	Deep vein thrombosis, pulmonary embolism (PE)*
	Pharmacist participation during daily rounds*	Frequency of clinical assessments*	Pressure ulcers, stress ulcers*
	Availability of a mechanical ventilation wearing protocol*	Availability of pain management protocols**	
	Presence of approved indications and contraindications for admission to, transfer within, and discharge from intensive care units*	Prevention of thromboembolic events*	
	Availability of a quality improvement strategy*	Prevention of ulcer formation*	
Acceptability	Is the ICU frightening or reassuring?	Is the conversation with relatives required and is the information about it recorded?	Are patients and relatives followed up to get their opinions and suggestions for improvement?
	What are the conditions for relatives (confidentiality of counseling, accommodation, overnight stay)?		
Efficiency	Avoiding waste in structure, equipment, personnel	Operational capacity, staffing levels, and related resources	Cost of similar cases
	Presence of protocols for daily sedation interruption*	Therapeutic Intervention Scoring System after the discharge*	Mortality rate*
	Are doctor’s recommendations entered into the computerized system?**	Unplanned extubation*	Patient/relative satisfaction level
	Frequency of delayed admissions and discharges from the department*	Frequency of blood component transfusions*	In-hospital mortality*
	Frequency of surgery cancellations and postponements*	Nutritional support*	
	Rate of repeated admissions to the intensive care unit*	Glucose level*	
		Discussion of autopsy results**	
		Number of patients discharged early*	
		Duration of mechanical ventilation**	
Access		Number of patients denied hospital admission due to lack of available beds	Subsequent outcomes of patients denied hospitalization due to bed unavailability
Equity		Evidence of selection bias in hospital admissions	Is there evidence of systematic bias influencing the outcomes?
Relevance	Considering other needs, is this service being delivered adequately?		What is the facility’s contribution to survival and health support, and which populations benefit most from its services?

Maxwell RJ. Dimensions of quality revisited: from thought to action. Quality in health care. 1992 Sep;1(3):171

*De Vos M, Graafmans W, Keesman E, Westert G, van der Voort PH. Quality measurement at intensive care units: which indicators should we use?. Journal of critical care. 2007 Dec 1;22(4):267–74

**additional aspects highlighted by the experts

Ethical approval for our study was obtained prior to data collection from the Ethics Commission of Communal Enterprise of Rivne region council “Yuri Semenyuk Rivne regional clinical hospital” (Protocol No. 29 MA/1411 dated November 14, 2024). This study was conducted in accordance with the ethical principles of the Declaration of Helsinki. Prior to completing the questionnaire, respondents provided their consent to the processing of personal data, thereby ensuring compliance with the ethical principles governing the study. In the beginning of the questionnaire, an obligatory consent field was included, ensuring that each respondent was fully informed about the purpose of the study and voluntarily agreed to participate.

In the introductory section of the online questionnaire, participants were informed that the study data would be anonymized.

**Data Collection Methodology.** An invitation to complete the questionnaire, along with the link to the Google Form, was distributed among doctors-anesthesiologists including heads of ICUs across various healthcare facilities in Ukraine. Initially, the dissemination was planned to conduct by means of a snowball sampling method through the researcher’s personal professional contacts in regional branches of the Ukrainian Association of Anesthesiology and Intensive Care. Each chief specialist of the regional branch was supposed to further distribute the invitation among anesthesiologists in intensive care units.

This approach encourages respondents who meet the inclusion criteria to forward the questionnaire to other potential participants. The use of the snowball sampling method is appropriate, as it includes the respondents possessing relevant experience and expertise in the subject area [40]. The combination of the snowball sampling method with a semi-structured survey enables both in-depth qualitative analysis of individual perceptions and quantitative evaluation of the significance of healthcare quality indicators [40]. The use of this method in our study was justified by the fact that direct access to respondents was limited.

However, during the data collection phase, respondent activity was low. Only ten responses were received within the first month after the questionnaire dissemination start.

The first ten respondents were contacted. The main barriers to participation in the study were identified. First, the large number of open-ended questions resulted in significant time needed to answer them, which may have decreased respondents’ motivation. Second, some questions were reported as difficult to understand, potentially affecting both the level of engagement and the quality of responses received.

It was decided to change the data collection method to Purposive sampling. This method involves the deliberate selection of research participants who possess relevant experience or expertise in the specific field and are capable of providing in-depth and meaningful responses to the research question [41].

To recruit respondents, we contacted the Department of Civil Protection and Public Health of the Rivne Regional State Administration with a request to send invitation letters to all the regional health departments of the Ministry of Health. Specifically, in Vinnytsia, Volyn, Donetsk, Zhytomyr, Zakarpattia, Zaporizhzhia, Ivano-Frankivsk, Kyiv, Kirovohrad, Luhansk, Lviv, Mykolaiv, Odesa, Poltava, Sumy, Ternopil, Kharkiv, Kherson, Khmelnytskyi, Chernivtsi, Chernihiv, Dnipropetrovsk, and Cherkasy regions.

The letter included a request to distribute the questionnaire among subordinate departments of healthcare institutions with intensive care units, specifying the purpose of the study. The letters were official in nature and were distributed with the appropriate acknowledgment of receipt. As a result, 114 completed questionnaires were collected, containing detailed responses to the open-ended questions.

According to the Public Organization “Ukrainian Association of Anesthesiology and Intensive Care” (UAAIT), the total number of anesthesiologists in Ukraine in 2024 was 6440, of whom 954 held positions of heads of intensive care departments^1^. In this study, 49 out of 114 responses (43%) were heads of departments, corresponding to a response rate (RR) of 5.14%. And 65 responses (57%) came from anesthesiologists, yielding an RR of 1.01%. At the same time, it was impossible to obtain the data on the number of patients who are treated annually in the responding regions because of the martial law in the country.

No duplicate or re-submitted questionnaires were identified. Furthermore, all received questionnaires contained complete responses to the open-ended questions. Consequently, all 114 completed questionnaires were included in the analysis.

Responses to the open-ended questions were initially categorized according to the established framework (see Table 1) and subsequently analyzed using descriptive statistics. This approach enabled summarization of the main sample characteristics, assessment of response distributions, and identification of key trends by systematizing and interpreting the data in a structured manner [42].

To categorize responses to the open-ended questions, thematic content analysis was applied, which enabled systematic classification and quantification of the frequency of key themes and concepts within the responses [43]. Consequently, it facilitated a deeper understanding of the context and content of the responses, uncovering both explicit and implicit themes. As a result, we obtained a more comprehensive and nuanced picture, allowing for improved interpretation of the results and identification of key patterns in participants’ responses [44].

The normality of the distribution of respondents’ age and work experience was assessed by the Kolmogorov-Smirnov test [45]. The analysis indicated a deviation from the normal (Gaussian) distribution, necessitating the use of nonparametric statistical methods [45]. Specifically, the Mann-Whitney U test was employed for further analysis, with results presented as median (Me) [interquartile range (IQR 25; 75)] [46].

To model the relationship between binary independent variables (gender, work experience, job title, English proficiency) and quality indicators as independent variables (for the wording see Tables 7 and 8), binary logistic regression analysis was employed. For this analysis, responses of “very important,” “important,” and “rather important” were coded as “important” = 0, while “not at all important” and “not important” were coded as “not important” = 1. Work experience was coded as “0” for up to 10 years and “1” for more than 10 years. Job title was coded as “0” for doctors and “1” for heads of departments. English proficiency was coded as “0” for “do not speak” and “basic level” and as “1” for “average,” “sufficient,” and “professional.” Statistical analysis was performed using IBM SPSS Statistics version 26, as well as Microsoft Excel and PowerPoint 2021.

## Results

Of the 114 completed questionnaires, 72.8% of questionnaires were filled in by male respondents, 27.2% by female. The average age of respondents was 46 years old, and the average experience was 22 years. 47.4% of the respondents worked in cluster institutions, 34.2% in general institutions, and 18.4% in above cluster institutions. The city council was the owner in 62.3%, the regional council in 26.3%, and the rural council in 11.4% of cases.

Table 2 shows the demographic characteristics of the study respondents in detail.

**Table 2:** Demographic characteristics of study respondents

Respondent characteristics	Value N = 114
Gender	
- Male	27.2%
- Female	72.8%
Age	
- Minimum	26
- Maximum	75
- Average	46.33
Job title	
- Anesthesiologist	56.1%
- Head of ICU	43.9%
Experience	
- Up to 10 years	31.6%
- More than 10 years	68.4%
English proficiency	
*- No*	57.0%
*- Yes*	43.0%
Institution Type*	
*- General*	34.2%
- Cluster	47.4%
- Above cluster	18.4%
Owner of the facility **	
- Rural councils	11.4%
- City council	62.3%
- Region (oblast) councils	26.3%

* *Ukrainian healthcare system has three level structure based upon territorial principle and the level of care*

1. **General medical facility** (primary care; basic secondary care, for example, pediatric care, surgery of medium complexity; covers one territorial unit)

2. **Cluster** (wider spectrum of care, higher level of diagnostic and treatment procedures; covers several territorial units – so called cluster; has specialized departments, for example, cardiology, urology, etc.)

3. **Above cluster** (the highest level of specialized care, including tertial care; covers a whole region, has the best equipment and personnel)

** medical facilities in Ukraine have so called owners, who also perform the functions of founders. Such owners are based on territorial principle and represented by:

1. rural councils (mostly primary care, general facility)

2. city councils (primary, secondary care)

3. region (oblast) councils (mostly cluster and above cluster facilities)

Questionnaires were filled in by respondents from 18 regions (oblasts) of Ukraine (out of 22 regions controlled by Ukraine), with the exception of Ivano-Frankivsk, Dnipropetrovsk, Kherson, and Poltava regions (Table 3). The reasons for thizs may vary, ranging from logistical and technical difficulties to differences in communication between regional health departments (HD) and local healthcare institutions. In some regions, interactions between HD and healthcare facilities are more active, facilitating better participation in such studies, whereas in others, these connections may be weaker. It is also important to note that doctors traditionally have low response rates to surveys. According to existing research, physician participation in online surveys rarely exceeds 20–30% [47].

**Table 3:** Percentage distribution of respondents by region

Region	Percentage of filled in questionnaires received (%)
Rivne	11.4
Kyiv	2.6
Lviv	8.8
Odesa	7.9
Chernihiv	7.9
Mykolaiv	1.8
Zhytomyr	2.6
Chernivtsi	1.8
Khmelnytskyi	0.9
Cherkasy	6.1
Vinnytsia	2.6
Zaporizhzhia	6.1
Sumy	0.9
Volyn	19.3
Kharkiv	3.5
Transcarpathia	10.5
Kirovohrad	3.5
Ternopil	1.8

*N = 22 regions that areunder the control of Ukraine

Table 4 presents respondents’ perceptions of healthcare quality. Answers to the question “What does quality in ICU mean to you?” were given mostly as an enumeration of keywords. Quality was primarily associated with equipment and resource monitoring (17.15%), effectiveness (17.15%), efficiency (12.97%), workforce management (12.55%), compliance to standards (11.31%), and timeliness (5.44%).

**Table 4:** Perception of the quality of medical care in the ICU among anesthesiologists

Quality attribute	Quotation
Equipment and resource monitoring = 41 (17.15%)	Providing necessary equipment and medicines, using modern methods and technologies, laboratory diagnostics, the latest technologies
Effectiveness *n* = 41 (17.15%)	Health improvement, quality of life of patients upon discharge from hospital, clinical outcomes, correct diagnosis, early rehabilitation of patients, incidence of complications, cost-effectiveness
Efficiency *n* = 31 (12.97%)	Treatment effectiveness, positive dynamics in treatment, length of stay, bed turnover, average bed stay, transfers to specialized departments, adequate patient pain management
Compliance to standards *n* = 27 (11.31%)	Compliance to global standards, clear implementation of SOPs, compliance to treatment protocols and patient routes
Timeliness *n* = 13 (5.44%)	Timely assistance
Work with personnel *n* = 30 (12.55%)	High level of training of all staff members, continuous training, team of highly qualified specialists, ratio of staff to patient number, feedback to/from staff, multidisciplinary approach
OTHER *n* = 56 (23.43%)	Clinical performance monitoring, equity, working conditions, incentives, patient-centeredness, patient safety, trust, credibility, accessibility

*N (number of quality attributes mentioned) = 239 (100%)

Table 5 illustrates how Ukrainian anesthesiologists perceive the key components of quality of care based on Maxwell’s criteria.

**Table 5:** Perception of healthcare quality and quality indicators used to assess the structure, process and outcome

Key indicators according to Maxwell	Perception	Measuring tools	Indicators for measurement
**Effectiveness**	**Structure –** availability of a recovery room; correspondence between the number of beds and the institution’s workload; availability of resources; professional experience (own or that of colleagues); degree of standardization. **Process –** average bed-day; regular audit procedures. **Outcome –** final treatment results; incidence of infectious complications; feedback from physicians and/or patients.	Audit, monitoring, patient surveys, work with complaints, communication, equipment, standardization, control	Urinary incontinence, length of stay, outcome, mortality, staff, cost-effectiveness, satisfaction, infectious complications, timeliness and speed
**Acceptability**	**Structure:** monitoring equipment and resources **Process:** patient-centricity, compliance, audit **Outcome:** result	Monitoring of equipment and resources, auditing, work with complaints, patient and family satisfaction surveys, laboratory and instrumental diagnostic methods, statistical analysis	Number of complaints from patients and their relatives, compliance to standards, rapid diagnostics
**Efficiency**	**Structure:** clinical indicators monitoring **Process:** compliance, patient-centeredness, audit **Outcome:** mortality, outcome	Monitoring of clinical indicators, standardization, audit, work with complaints, treatment outcome indicators, analysis of mortality indicators, indicator of workload on medical personnel, assessment of the use of medical equipment	Number of patients with chronic pain, number of patients with chronic postoperative pain, improvement and stabilization of the patient’s condition according to clinical and physical indicators, objective patient status, absence of patient complaints, quality and patient safety indicators
**Access**	**Structure:** monitoring equipment and resources, working with personnel **Process:** no barriers, equality, waiting times, compliance to standards, audit **Outcome:** free of charge, compliance with standards	Monitoring equipment and resources, working with personnel, auditing, monitoring financial barriers	Availability of medical supplies, medical instruments, medical equipment, an inventory of material and technical resources, number of qualified personnel, absence of obstacles, equality, waiting time, compliance to standards, provision of the services free of charge, analysis of treated patients, capability to perform examinations and treatment of patients at a modern level on a 24-hour basis, protocols.
**Equity**	**Process:** waiting time, empathy, equipment and resource monitoring, compliance, patient-centeredness, audit **Outcome:** result	Monitoring of equipment and resources, standardization, auditing, monitoring of the level of availability of services for different population groups	Providing full medical care and empathy for each patient, assessing the availability of medical care, waiting time, lack of discrimination, availability of clinical guidelines, socio-demographic analysis, transfer to a specialized department
**Relevance**	**Process:** Audit, compliance to standards **Outcome:** outcome	Standardization, expert assessment of medical history, audits of clinical processes, monitoring of clinical effectiveness	Adherence to the patients’ routes and clinical guidelines, compliance to protocols, treatment outcome, general condition of patients

The data presented in the table indicate that anesthesiologists evaluate quality in intensive care units through the dimensions of structure, process, and outcome, with particular emphasis on effectiveness and resource availability. Efficiency and accessibility primarily relate to the availability of equipment and staff performance, whereas acceptability and equity reflect patient-centeredness and compliance to standards. Compliance to standards serves as a key quality indicator, highlighting the critical role of a robust quality management system.

Table 6 presents the results of the binary regression. The model was significant only for the indicator “Satisfaction of the patient’s relatives with the patient’s stay in the ICU” (sig 0.002). We found a statistically significant relationship (p˂0.05) between the indicator and the independent variable “Owner”.

**Table 6:** Binary regression results

	Patient’s family satisfaction “0” Not important 16.5% “1” Important 83.5%		
	Exp(B)	95% C.I. for Exp(B)	
**Independent variables**		**Lower**	**Upper**
Age	0.981	0.914	1.053
Work experience	5.778	0.821	40805
Job title	0.475	0.122	1.858
Gender	0.306	0.52	1.786
Type of institution	0.420	0.158	1.117
Owner	**0.280**	**0.083**	**0.948**
English proficiency	1.701	0.454	6.364
Constant	1356.259		
Nagelkirk’s R-squared	0.310		

A statistically significant relationship of the independent variable “Owner” was also observed with the indicator “Availability of pain management protocols” (sig 0.034. Exp(B) 68.506, confidence interval 1.381–3399.216, constant 5.760, Nagelkirk R-square 0.377), however, the model in this case was not statistically significant (sig 0.206).

A statistically significant association was observed between the independent variable “Job title” and the indicator “Patient opinions and wishes” (*p* = 0.025; Exp(B) = 3.924; 95% CI: 1.057–14.563; constant = 27.655; Nagelkerke R^2^ = 0.169). However, the model in this case was also not statistically significant (*p* = 0.09).

Tables 7 and 8 demonstrate non-clinical and clinical quality indicators that are important for the respondents.

**Table 7:** Importance of the non-clinical quality indicators

Non-clinical indicator (N = 115)	Value range	Valid percentage
Doctor -to-patient ratio	“0” no “1” yes	1.7% 98.3%
Nurse-to-patient ratio	“0” no “1” yes	0% 100%
Assistant-to-patient ratio	“0” no “1” yes	2.6% 97.4%
Availability of necessary equipment	“0” no “1” yes	1.7% 98.3%
Pharmacist participation in daily rounds	“0” no “1” yes	40.9% 59.1%
Approved admission and discharge criteria for ICU	“0” no “1” yes	1.7% 98.3%
Strategy for improving the quality of medical care in intensive care units	“0” no “1” yes	1.7% 98.3%
Does the department make the patient anxious or calm?	“0” no “1” yes	10.4% 89.6%
Shift duration	“0” no “1” yes	1.7% 98.3%
Conditions for relatives of patients	“0” no “1” yes	19.1% 80.9%
Patients’ opinions and wishes	“0” no “1” yes	17.4% 82.6%
Compliance to protocols	“0” no “1” yes	3.5% 96.5%
Patient satisfaction with the stay in the ICU	“0” no “1” yes	10.4% 89.6%
Satisfaction of the patient’s relatives with the patient’s stay in the ICU	“0” no “1” yes	16.5% 83.5%
Significance of empathy shown by medical staff	“0” no “1” yes	4.3% 95.7%

**Table 8:** Clinical quality indicators that are important for ICU anesthesiologists in Ukraine

Clinical indicator (N = 115)	Value range	Valid percentage
Prevention of thromboembolism	“0” no “1” yes	0.9% 99.1%
Prevention of ulcer formation	“0” no “1” yes	1.7% 98.3%
Survival rates in comparison with similar cases in similar departments	“0” no “1” yes	2.6% 97.4%
Complications and infection rates	“0” no “1” yes	1.7% 98.3
Control of ventilator-associated pneumonia	“0” no “1” yes	1.7% 98.3
Deep vein thrombosis/Pulmonary embolism	“0” no “1” yes	0.9% 99.1%
Pressure ulcer prevention	“0” no “1” yes	1.7% 98.3%
Mechanical ventilation wearing protocol	“0” no “1” yes	0% 100%
Compliance to protocols	“0” no “1” yes	3.5% 96.5%
Compliance to evidence-based requirements	“0” no “1” yes	0.9% 99.1%
Open or closed TBT suction systems	“0” no “1” yes	1.7% 98.3%
Frequency of clinical case reviews	“0” no “1” yes	2.6% 97.4%
Availability of pain management protocols	“0” no “1” yes	2.6% 97.4%

Among the most important clinical quality indicators, such indicators as “Mechanical ventilation weaning protocol” (100%), “Prevention of thromboembolism” (99.1%), “Prevention of ulcer formation” (98.3%), “Control of ventilator-associated pneumonia” (98.3%), and “Compliance to protocols” (96.5%) were mentioned. Among the non-clinical indicators, the “Nurse-to-patient ratio” (100%) was identified as the most important. In contrast, “Pharmacist participation in daily rounds” (59.1%), the “Provision of conditions for patients’ relatives” (80.9%) and the “Patient satisfaction”, “Satisfaction of patients’ relatives” (89.6% and 83.5%, respectively) were rated moderately or even low. For example, “Participation of pharmacist in daily rounds” was “very important” for 6.1% of respondents and “not important” for 30.7%.

Table 9 presents opinions of the respondents on staff-to-patient ratios and shift duration. Patient to staff ratio of 1:3 was most frequently reported as optimal. Regarding shift duration, the 8-hour workday was most mentioned (47.1%), which may reflect an intention to minimize staff fatigue and prevent overload.

**Table 9:** Optimal staff-to-patient ratios and shift duration

Indicator	Value	%
Doctor to patient ratio	1:3	**26.39%**
	1:6	25.69%
	1:4	23.61%
	1:5	11.81%
	1:2	4.86%
	1:8	4.17%
	1:7	2.78%
	1:1	0.69%
Nurse to patient ratio	1:3	**42.42%**
	1:2	28.03%
	1:1	15.91%
	1:4	9.85%
	1:5	2.27%
	1:6	1.52%
Assistant to patient ratio	1:3	**32.43%**
	1:6	19.82%
	1:4	14.41%
	1:2	12.61%
	1:5	12.61%
	1:1	5.41%
	1:8	2.70%
Shift duration	8 hours	**47.13%**
	12 hours	24.14%
	24 hours	14.41%
	6 hours	8.05%
	16 hours	3.45%
	72 hours	1.15%

## Discussion

This study focused on the perceptions of quality by medical doctors – anesthesiologists who work in intensive care units (ICU) in Ukraine. The discussion strategy is built around three core issues revealed by the research: how Ukrainian ICU anesthesiologists perceive the quality of care in relation to Maxwell’s framework; what quality indicators are important for the anesthesiologists; how these findings compare with evidence and practice from other countries.

Quality monitoring and assessment by means of indicators assure healthcare quality, benchmark in time between different medical facilities, help in decision making and setting priorities, support reporting as well as improve quality [48]. Indicators show how well healthcare professionals and providers function to satisfy the needs of their patients [48]. At the same time, it is not enough to use just the indicators to assess quality (Mainz, 2003a). Use of different valid and reliable assessment tools will show the level of quality clearer and lead to systematic improvement of healthcare quality [49]. Moreover, the perception of quality indicators among those who perform the assessment predisposes their choice and further use in the routine practice [18].

The results of our study show that the perception of healthcare quality among anesthesiologists in ICU in Ukraine are partially in consistence with the Maxwell’s framework. The highest level of correspondence was observed in the assessment of outcome domain. The perception of outcome effectiveness in ICU corresponds Maxwell’s criteria in 83% of responses. This shows that the medical doctors anesthesiologists in ICU in Ukraine perceive quality as outcome quality focusing on clinical outcomes and positive patient dynamics.

The lowest level of correspondence was observed in process and structure domains. This might be explained by the fact that under the clinical work overload and limited resources medical doctors tend to focus on directly measurable and critical indicators such as medical personnel and patient ratio, workload, access to the equipment and protocols. For example, all the respondents (100%) associated the acceptability of the structure with the monitoring of the equipment and resources. The same was true for acceptability of process and outcome – the majority of perceptions were expressed by means of audit or clinical associations.

These might have several explanations. As to the authors’ experience, clinical professionals, specifically doctors-anesthesiologists tend to focus on clinical or so-called technical quality aspects (for example, equipment, treatment outcomes, compliance to clinical guidelines). At the same time, such quality aspects as equality, acceptance or compliance to patients’ expectations are left outside their scope of attention.

Another explanation might be that the weak integration of quality assessment tools (for example, audit, standardization, processes assessment) into the practice might result in their perception by the medical doctors as external quality component. Also, the context of the system of low resources, like in Ukraine pushes healthcare professionals to prioritize basic needs – resources, equipment, minimal criteria of the outcome.

These observations are confirmed by other studies. Specifically, the study Bastos et al. [50] analyzed the effectiveness of intensive care units in low-resource countries. The database of the randomized clinical research CHECKLIST-ICU with more than six thousand patients in 118 ICUs in Brazil was analyzed. Influence of the structural and process factors on clinical effectiveness was studied. The results showed that the organization of care, compliance to clinical guidelines and interdisciplinary interactions of the personnel play the critical role in clinical outcomes improvement even under the conditions of low resources. These processes often have low integration into the routine practice, which also confirm our observation about the underrating of managerial quality tools among the doctors-anesthesiologists in Ukraine.

According to the modified Delphi study by Yee & Tarshis [51], conducted in Canada with 14 anesthesiologist experts from different regions of the country, 52% of indicators referred to outcomes (e.g., rate of intraoperative and postoperative complications, adequacy of pain control, patient satisfaction, infection and thrombosis prevention), 35% to processes (e.g., adherence to clinical protocols, safety checklists, interprofessional communication, frequency of audits), and 12% to structure (e.g., staff-to-patient ratio, equipment availability, monitoring systems). This distribution demonstrates that even in high-income settings, anesthesiologists predominantly associate quality with clinical outcomes, while organizational and structural components of quality management receive comparatively less attention.

A similar pattern is observed in the United States of America. According to the analysis by Hyder et al. [52], which reviewed all 637 performance measures endorsed by the National Quality Forum (NQF), 6 indicators (1%) were specific to anesthesiology, the majority of relevant measures for anesthesiologists were jointly attributable and predominantly outcome-focused—73% of these related to mortality or major postoperative complications (e.g., cardiac events, respiratory complications). This demonstrates that even in a highly standardized evaluation environment, quality in anesthesiology is largely conceptualized through clinical outcomes, with limited development of specialty-specific process or structure indicators.

In comparison with our findings from Ukraine - where anesthesiologists also prioritize outcome indicators and place less emphasis on managerial, patient-centered, or structural dimensions - this suggests a broader international trend of outcome-oriented perception of quality within the specialty, though in Ukraine it is additionally reinforced by resource limitations and weak integration of formal quality management tools. At the same time, we did not include the ICU nurses in our study, who might be more focused on the process.

Open questions about the importance of the quality attributes in our study were answered using the key words naming both assessment tools (for example, audit, monitoring) and quality indicators used for such an assessment (for example, urinary incontinence). This might show that the doctors-anesthesiologists in Ukraine do not clearly differentiate the notions of “tool” and “indicator” or the formulation of the question in a questionnaire was unclear for the respondents.

The limited introduction of formalized quality assessment into Ukrainian ICUs might be an explanation. Audit, quality surveys or analysis of the indicators are not routine or standardized practice. Besides, part of the respondents might refer to their own clinical experience and might not differentiate enough between the quality management terms. Similar observation was also made by Anufriyeva et al. [22] who hypothesized that such lack of differentiation of the terms might be explained by the limited access to literature on healthcare quality because of low level of knowledge of the English language among medical doctors in Ukraine. This hypothesis was not confirmed in case of doctors-anesthesiologists in our study. Performed by us binary logistic regression analysis showed no statistically significant relationship between the binary independent variable “English proficiency” and the dependent variables - quality indicators of medical care in ICU.

In addition to assessing perceptions of care quality, the questionnaire also included items related to anesthesiologists’ knowledge and understanding of quality management principles. Although this aspect was not the primary focus of the study, the findings indicate varying levels of awareness and familiarity with quality management concepts among respondents. These results suggest a need for further education and integration of quality management principles into professional training and hospital governance practices.

Frequency of choice of certain clinical and non-clinical indicators also shows doctors-anesthesiologists’ priorities. Specifically, the importance of the medical doctors/nurses-to-patients ratio was expressed by almost 100% respondents, which is confirmed by the study Bastos et al. [50], that shows the crucial importance of personnel, clear structuring of the processes and access to the approved standards of patients safety.

The study Aiken et al. [53], conducted in the USA among more than 7,000 nurses who worked in different hospitals of the country, showed that the low nurses-to-patients ratio is associated with the higher patients mortality rate and higher level of professional burnout and work dissatisfaction in nurses.

The study Needleman et al. [54], also conducted in the USA among 5,000 nurses in hospitals confirmed the observations of the Aiken et al. [53] study adding to it the observation that the lack of personnel is also connected with the increase of the number of clinical mistakes and worsening of healthcare quality.

Our research also adds to the explanation as to why these indicators raise interest and discussions between the doctors-anesthesiologists who face overload in their clinical practice.

High level of importance was also observed for the clinical guidelines, specifically “Mechanical ventilation wearing protocol” 100%; “Deep Vein Thrombosis/Pulmonary Embolism” 99.1%; “Complications and infection rates” – over 98%. This might indicate the focus on evidential clinical practice in ICU. Compliance to clear standards helps to reduce variations in medical care and improve the patients’ prognosis [55].

Instead, patient-oriented indicators such as “Patient satisfaction” (89.6%), “Conditions for relatives” (80–82.6%), “Medical personnel empathy” (95.7%) have high importance rates but are not prioritized like clinical or structural ones. It might be connected with the fact that in the limited resources healthcare systems subjective indicators of patient or patients’ relatives experiences are perceived as less relevant under the conditions of time deficiency, lack of personnel or material resources [56].

In a narrative review titled “Using quality indicators in anaesthesia: feeding back data to improve care” [57] explored how anesthesiologists in United Kingdom measure and report quality of care. The study highlighted that metrics collected during the immediate post‑anesthetic recovery period - such as patient temperature, patient‑reported quality of recovery, pain and nausea - provide potentially useful information yet are not routinely fed back. It emphasized that effective quality indicator systems must include timely, credible, tailored and continuous feedback, rather than mere data collection.

A recent European study by Wacker et al. [58] surveyed 37 national anesthesiology societies (NAS) to analyze peri-operative quality indicators promoted across Europe (including Belgium; Czech Republic; Denmark; Estonia; Finland; France; Germany; Italy; Netherlands; Norway; Poland; Portugal; Romania; Spain; Sweden; Switzerland; United Kingdom and Ireland). The study identified 163 distinct quality indicators, of which 41.7% pertained to structure, 35.6% to safety, 20.9% to appropriateness, 16.6% to prevention, and only 3.7% addressed patient-centered care. Only 12 of the 37 societies (32%) had their own set of quality indicators, and in 6 countries (16.2%) their use was mandatory. This highlights that, similar to Ukraine, European anesthesiologists focus largely on structural and safety aspects, while patient-centered outcomes and systematic feedback for improvement are less emphasized.

Certain indicators such as “Pharmacist participation in daily rounds” (59.1%) or “Frequency of clinical case reviews” (97.4%) have less discussions in the professional environment in Ukraine. This might be explained by the step-by-step but not fully integrated into the structure of ICU understanding of the role of multidisciplinary approach and continuous learning. Also, this might indicate the variation in local practices, absence of clear guidelines or legislative support of such processes in medical management.

“Compliance to standards” and “Audit” were often mentioned by the Ukrainian anesthesiologists as substitutes or additions to the attributes offered by Maxwell, specifically in assessment of acceptability and outcome. This might point to the necessity to adapt the classical approaches to quality management to the local context in which the notion of effectiveness is tightly connected with legal compliance and control.

Among tested models of binary logistic regression only the model for the relationship between the dependent variable “Satisfaction of the patient’s relatives with the patient’s stay in the ICU” and independent variable “Owner” turned to be statistically significant. In the context of this study, the variable “Owner” partially indicates the type of settlement, where the medical facility is situated (urban, rural). Such an association might show that in urban hospitals the issue of feedback from patients’ relatives is perceived as more important in comparison with the rural medical facilities [59]. It might be explained by higher expectations urban citizens have, more active participation of patients and their relatives in the treatment process, easier access to leaving their feedback as well as more openness to service-oriented approach.

Besides, in big cities state medical facilities try to compete with the private ones for patients who require certain healthcare services. Thus, higher attention to “satisfaction” might be explained by more intensive competing environment in the cities. In healthcare facilities located in rural or less urban areas, attention to the patient-oriented and service interactions attributes might be lower because of personnel shortage, limited resources and focus of the personnel on basic clinical indicators of effectiveness.

A recent study by Mansoor et al. [60] in Pakistan examined the efficiency of post‑anesthesia care unit (PACU) discharges from a non-clinical audit perspective. The authors found that delays in PACU discharge were often due to organizational, administrative, or resource-related factors rather than clinical performance. They emphasized the importance of monitoring structural and process aspects—such as staff availability, equipment readiness, and administrative support—as integral components of quality management, alongside clinical outcomes. Similar to the Pakistani study, the lack of systematic integration of process and structural assessments in Ukrainian ICUs may limit opportunities for improvement, suggesting that broader incorporation of organizational and administrative quality measures could enhance care efficiency and overall quality.

In our study the low number of statistically significant models might also indicate that quality perceptions in ICU is a multifactor phenomenon, which is only partially conditioned by professional or demographic characteristics. Bigger role might be played by local clinical traditions, informal practices, managerial autonomy and communication culture in the team. This observation is supported by research focused on the influence of organizational culture on the behavior of medical personnel. For example, Larson et al. [61] show that microclimate in the department, interdisciplinary cooperation and support from the management are the key factors in creating the attitudes towards non-clinical aspects of quality, specifically those that are connected with patients’ experience of stay in a medical facility.

Among the limitations of our study the low participation rate should be mentioned, specifically among the doctors-anesthesiologists who are not holding the managerial positions. This could limit the representativeness of the study. Some respondents mentioned that the questionnaire was filled in by the department collectively and not by separate healthcare professionals. This could create certain bias in interpretation of the individual professional experience. One more factor which could limit enrollment into the study was complexity and volume of the questionnaire, specifically a lot of open-ended questions that required much time to be filled in. Additionally, the study was conducted under wartime conditions, which required certain adaptations of the research methods and may have influenced data collection and implementation processes.

## Conclusions

In conclusion, our research provides new insights into perception of healthcare quality and importance of quality indicators for its assessment among the medical doctors of specialized care, namely doctors-anesthesiologists in intensive care units in Ukraine. We identified fifteen non-clinical and thirteen clinical indicators and ranged their importance by those who directly use them in their routine practice.

In comparison with the general practitioners focusing on process quality, the focus of doctors-anesthesiologists lies in outcome quality. But little consensus about healthcare quality remains to be an issue for this group as well. As does the necessity to develop and promote a national quality policy and a national quality strategy. War in Ukraine complicates this process but at the same time makes this need even more acute as the pressure in ICU increases with the increased number of patients and complexity of war traumas. [62, 63]

## Electronic supplementary material

Below is the link to the electronic supplementary material.

Supplementary Material 1

## Data Availability

Available upon request.

## Footnotes

1 The data are not publicly available. The researcher contacted the UAAI to obtain information on the number of practicing anesthesiologists in Ukraine.
