# Supplementary material for: Perceptions of healthcare quality indicators by anesthesiologists in intensive care departments in Ukraine
Source: BMC Health Serv Res. 2025 Dec 23;26:126. doi: 10.1186/s12913-025-13922-z (PMC12838420; doi:10.1186/s12913-025-13922-z)
Supplement: Supplementary file 1 — Supplementary Material 1 [file 12913_2025_13922_MOESM1_ESM.docx]

**Appendix 1. Interview Guide**

The aim of this study is to explore the perceptions of healthcare quality indicators among doctors-anesthesiologists in intensive care units.

The study is conducted for educational purposes within the course *“Healthcare Management”* at the National University of “Kyiv-Mohyla Academy.”

Your expertise is very valuable for us. Participation is voluntary; you may withdraw at any stage. All information is confidential. Anonymized results may be discussed with researchers, representatives of authorized state bodies, and published while maintaining confidentiality. No financial compensation is provided.

**Consent**

I give my consent as a data subject to the processing of my personal data by the organizers of this survey for scientific analysis.

- Yes

**Block 1. Perception of Quality and Assessment Tools**

1. Please answer the question: *“What does quality in ICU mean to you?”*
2. Evaluation of **structure, process, and outcome**:
   - Name the tools you use to assess **structure**: __________________
   - Name the tools you use to assess **process**: ___________________
   - Name the tools you use to assess **outcome**: _________________
3. Perception of six aspects of healthcare quality (Robert Maxwell model):

3.1. *What does Effectiveness mean to you?* ___________

- - Tools for assessing effectiveness: __________________

3.2. *What does Acceptability mean to you?* ___________

- - Tools for assessing acceptability: __________________

3.3. *What does Efficiency mean to you?* ___________

- - Tools for assessing efficiency: __________________

3.4. *What does Accessibility mean to you?* ___________

- - Tools for assessing accessibility: __________________

3.5. *What does Equity mean to you?* ___________

- - Tools for assessing equity: __________________

3.6. *What does Relevance mean to you?* ___________

- - Tools for assessing relevance: ______________

**Block 2. Importance of Quality Indicators**

Please rate the importance of the following indicators using the following scale: Not at all important / Not important / Rather important / Important / Very important

2.1. Doctor-to-patient ratio

2.2. Nurse-to-patient ratio

2.3. Assistant-to-patient ratio

2.4. Availability of necessary equipment

2.5. Pharmacist participation during daily rounds

2.6. Availability of a weaning protocol from mechanical ventilation

2.7. Approved admission and discharge criteria for ICU

2.8. Availability of a quality improvement strategy

2.9. Shift duration

2.10. Compliance to protocols

2.11. Compliance to evidence-based requirements

2.12. Open or closed TBT suction systems

2.13. Frequency of clinical case reviews

2.14. Availability of pain management protocols

2.15. Thromboembolism prevention

2.16. Pressure ulcer prevention

2.17. Comparison of survival rates with similar ICUs

2.18. Complications and infection rates

2.19. Ventilator-associated pneumonia

2.20. Deep vein thrombosis / Pulmonary embolism

2.21. Pressure ulcers, stress ulcers

2.22. Does the ICU environment make the patient anxious or calm?

2.23. Conditions for relatives (privacy of consultations, accommodation, overnight stay)

2.24. Requirement of conversations with relatives and documentation of such communication

2.25. Follow-up communication with patients/relatives to receive feedback and suggestions

**Block 3. Interpretation and Perception of Indicators**

3.1. What do you consider to be the optimal physician-to-patient ratio? __________

3.2. What do you consider to be the optimal nurse-to-patient ratio? __________

3.3. What do you consider to be the optimal assistant-to-patient ratio? ________

3.4. Who should approve necessary equipment for ICU care? __________

3.5. Who should develop the ICU quality improvement strategy? __________

3.6. What should be the optimal duration of the shift? __________

3.7. Who should compare survival rates across ICUs? __________

3.8. Who should assess whether an ICU is frightening or reassuring? __________

3.9. Importance of patient satisfaction with ICU stay

Not at all important / Not important / Rather important / Important / Very important

3.10. Importance of relatives’ satisfaction with ICU stay

Not at all important / Not important / Rather important / Important / Very important

3.11. Importance of empathy of medical staff for quality of care

Not at all important / Not important / Rather important / Important / Very important

3.12. Which **non-clinical indicators** of quality are:

- Most important to you? __________________
- Most used in your work? __________________
- Most informative? __________________

3.13. Which **clinical indicators** of quality are:

- Most important to you? __________________
- Most used in your work? __________________
- Most informative? __________________

**Block 4. Participant Information**

4.1. Your age: __________

4.2. Years of work experience: __________

4.3. Your job title:

- Head of ICU
- Anesthesiologist

4.4. Your gender: __________

4.5. Type of facility where you work:

- General medical facility
- Cluster
- Above cluster

4.6. Facility is owned by:

- Rural council
- City council
- Regional council
- Private medical facility

4.7. Your level of English proficiency:

- Do not speak
- Basic
- Average
- Sufficient
- Professional

**Thank you for your time and valuable input!**
